# Supplementary material for: Spin dynamics, loop formation and cooperative reversal in artificial quasicrystals with tailored exchange coupling
Source: Commun Phys. 2023 Jul 29;6(1):193. doi: 10.1038/s42005-023-01310-0 (PMC11041715; doi:10.1038/s42005-023-01310-0)
Supplement: Supplementary file 1 — Supplementary Information [file 42005_2023_1310_MOESM1_ESM.pdf]

Supplementary Information

Spin Dynamics, Loop Formation and Cooperative Reversal in Artificial Quasicrystals with Tailored Exchange Coupling

Vinayak Shantarm Bhat, Sho Watanabe, Florian Kronast, Korbinian Baumgaertl, and Dirk Grundler**

The document contains further analyzed data that shows the as-grown state, initial state (after applying the maximum field, that is, -52 mT), and the final state (after applying the maximum field in the reverse direction, that is, +52 mT) for Penrose P2, P3, and Ammann quasicrystal lattices.

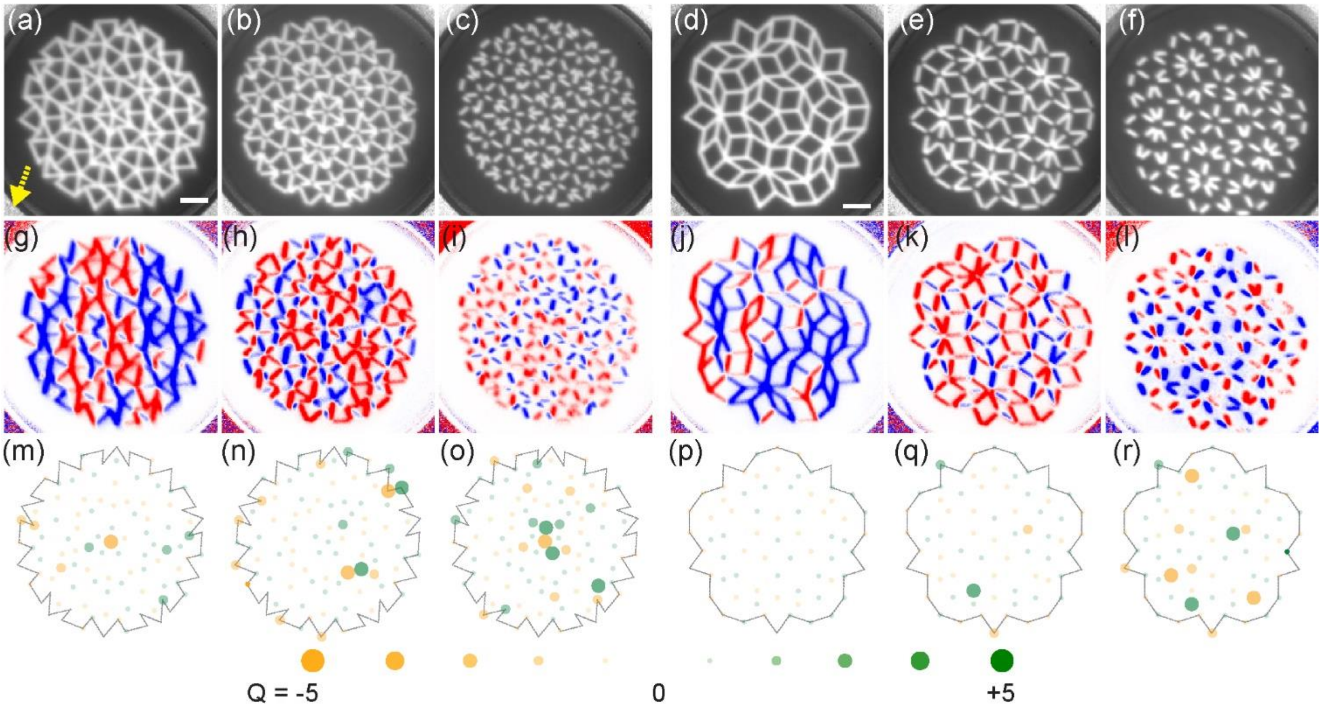

Supplementary Figure 1: PEEM topography image for (a) P2A, (b) P2B, (c) P2C, (d) P3A, (e) P3B, and (f) P3C, respectively in the as-grown state. Bright (dark) regions correspond to magnetic (non-magnetic) regions. The scale bar corresponds to 1 μm. The dotted downward yellow colored arrow represents the X-ray direction. (g) - (l) corresponding XMCD data of topography images shown in (a) – (f). Blue (red) colors represent magnetization parallel (opposite) to the X-ray direction. Notice the weak contrast of nanobars whose orientation deviates from the X-ray direction. (m) – (r) CM analysis of XMCD experimental data shown in (g) – (l). The green and orange filled circles at the vertices represent positive and negative vertex charges, respectively. Here the circles with smallest and largest diameter represent charge $Q = 0$ and $Q = 5$, respectively.

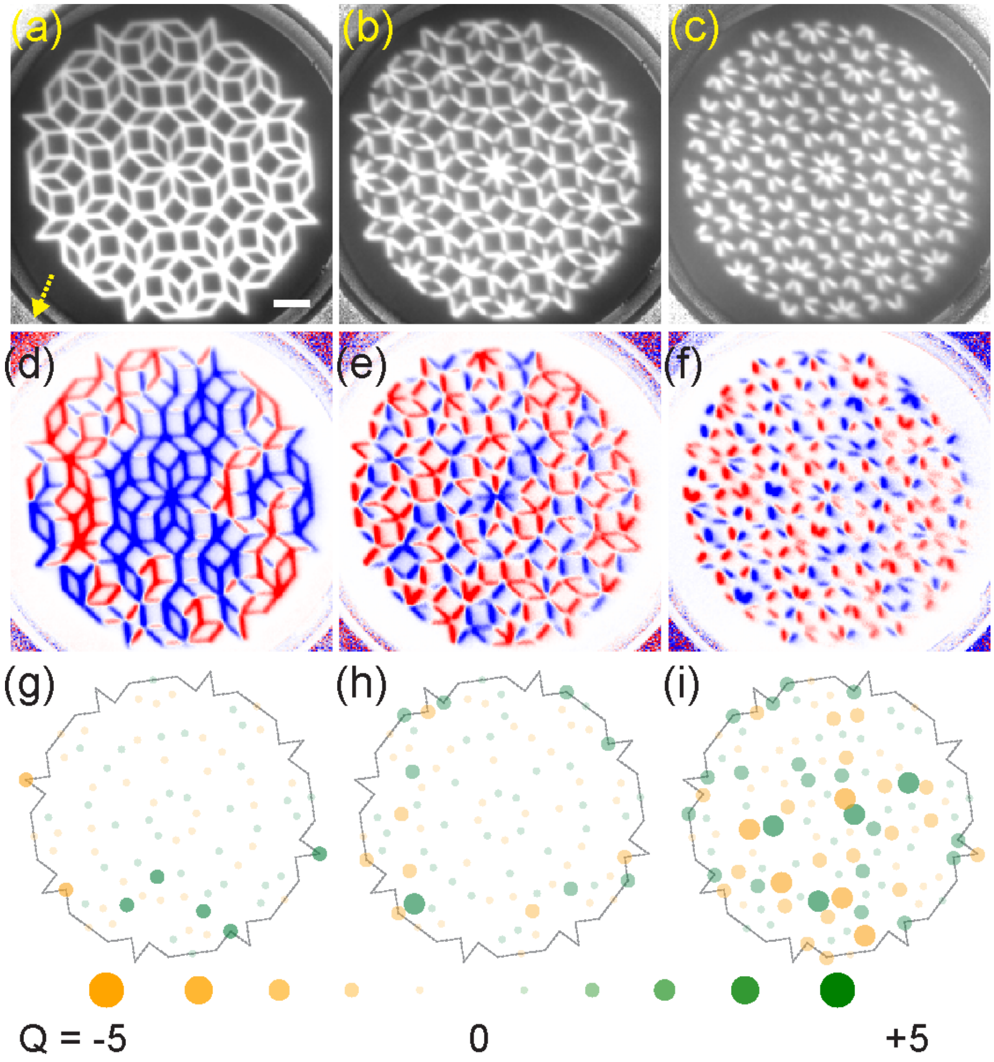

Supplementary Figure 2. PEEM topography image for as-grown state samples that were nominally identical to (a) ATA, (b) ATB, and (c) ATC, respectively. Bright (dark) regions correspond to magnetic (non-magnetic) regions. The scale bar corresponds to 1 µm. The dotted downward yellow colored arrow represents the X-ray direction. (d) - (f) corresponding XMCD data of topography images shown in (a) – (c). Blue (red) colors represent magnetization parallel (opposite) to the X-ray direction. (g) – (i) CM analysis of XMCD experimental data shown in (d) – (f). The green and orange filled circles at the vertices represent positive and negative vertex charges, respectively. Here the circles with smallest and largest diameter represent charge $Q = 0$ and $|Q| = 5$, respectively.

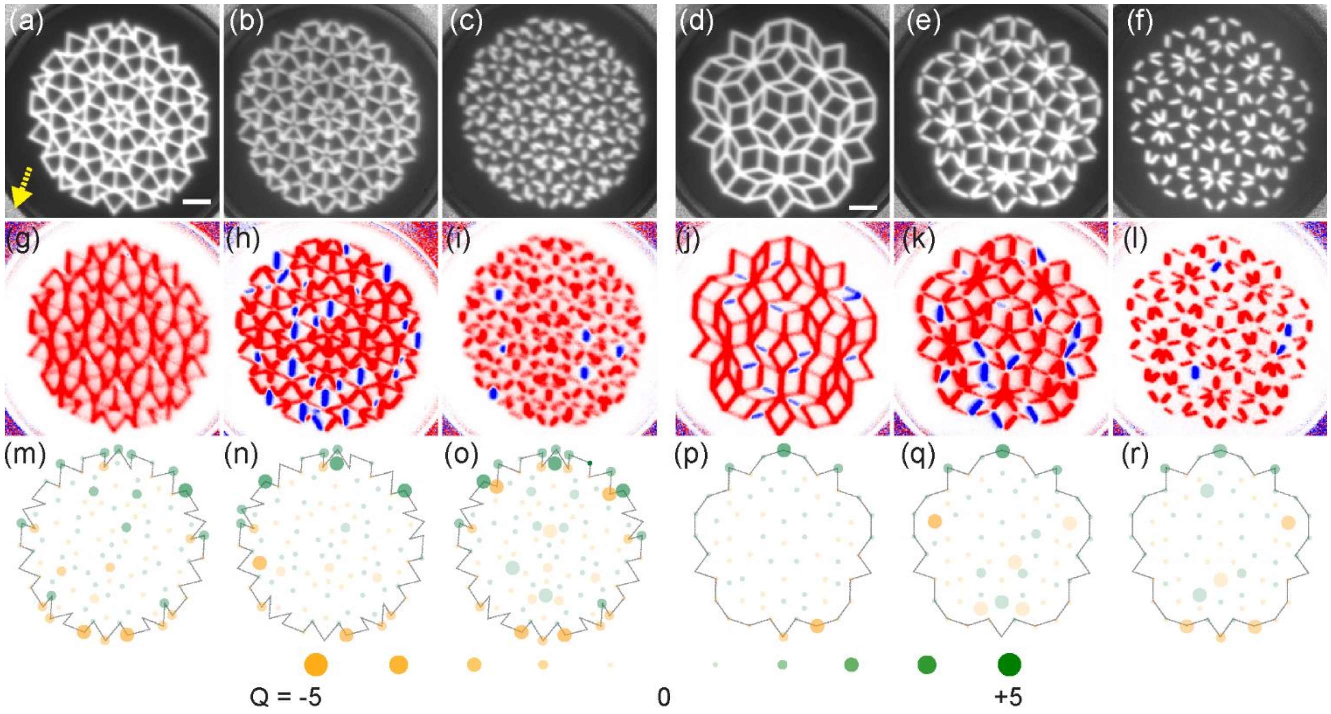

Supplementary Figure 3. PEEM topography image for (a) P2A, (b) P2B, (c) P2C, (d) P3A, (e) P3B, and (f) P3C, respectively in the initial state corresponding to -52 mT → 0 mT. Bright (dark) regions correspond to magnetic (non-magnetic) regions. The scale bar corresponds to 1 μm. The arrow in (a) represents the X-ray direction and the magnetic field direction. (g) - (l) corresponding XMCD data of topography images shown in (a) – (f). Blue (red) colors represent magnetization parallel (opposite) to the X-ray direction. Blue color indicates a reversed nanobar. Notice the weak contrast of nanobars whose orientation deviates from the X-ray direction. (m) – (r) CM analysis of XMCD experimental data shown in (g) – (l). The green and orange filled circles at the vertices represent positive and negative vertex charges, respectively. Here the circles with smallest and largest diameter represent charge $Q = 0$ and $|Q| = 5$, respectively.

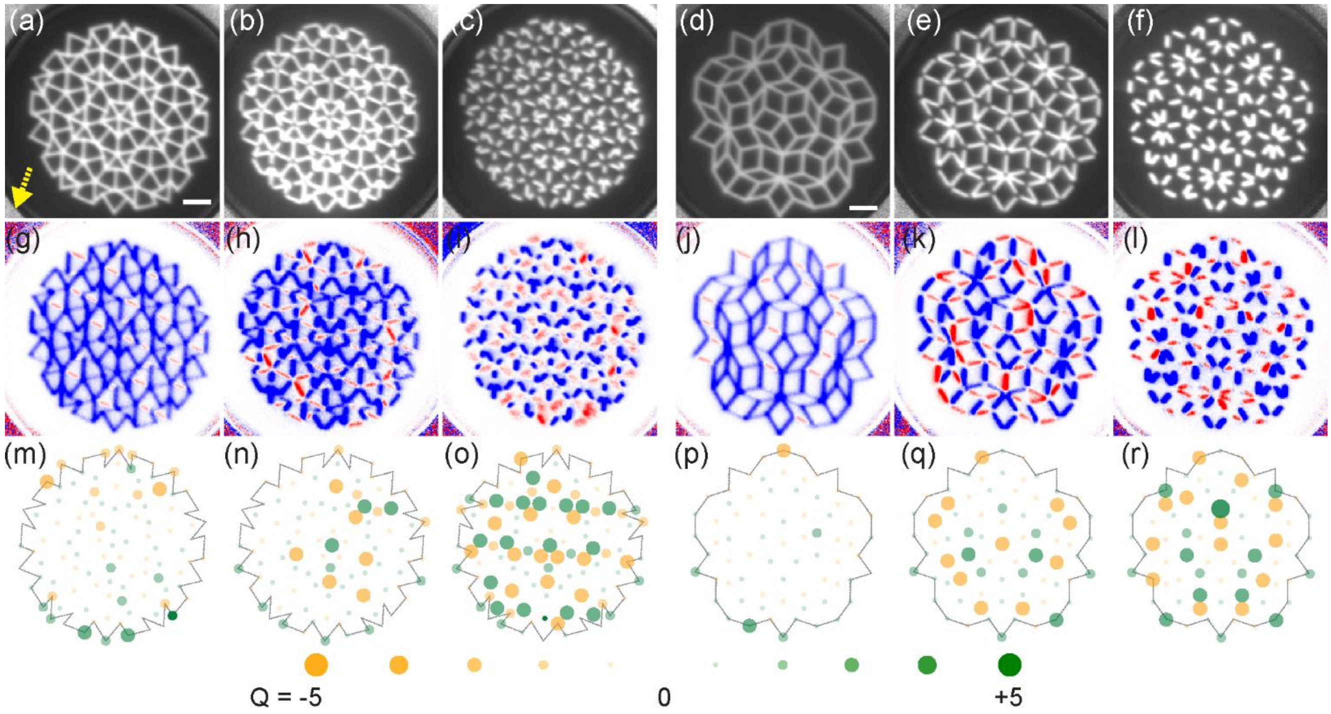

Supplementary Figure 4. PEEM topography image for (a) P2A, (b) P2B, (c) P2C, (d) P3A, (e) P3B, and (f) P3C, respectively in the final state corresponding to +52 mT → 0 mT. Bright (dark) regions correspond to magnetic (non-magnetic) regions. The scale bar corresponds to 1 µm. The arrow in (a) represents the X-ray direction and the magnetic field direction. (g) - (l) corresponding XMCD data of topography images shown in (a) – (f). Blue (red) colors represent magnetization parallel (opposite) to the X-ray direction. Blue color indicates a reversed nanobar. (m) – (r) CM analysis of XMCD experimental data shown in (g) – (l). The green and orange filled circles at the vertices represent positive and negative vertex charges, respectively. Here the circles with smallest and largest diameter represent charge $Q = 0$ and $|Q| = 5$, respectively.

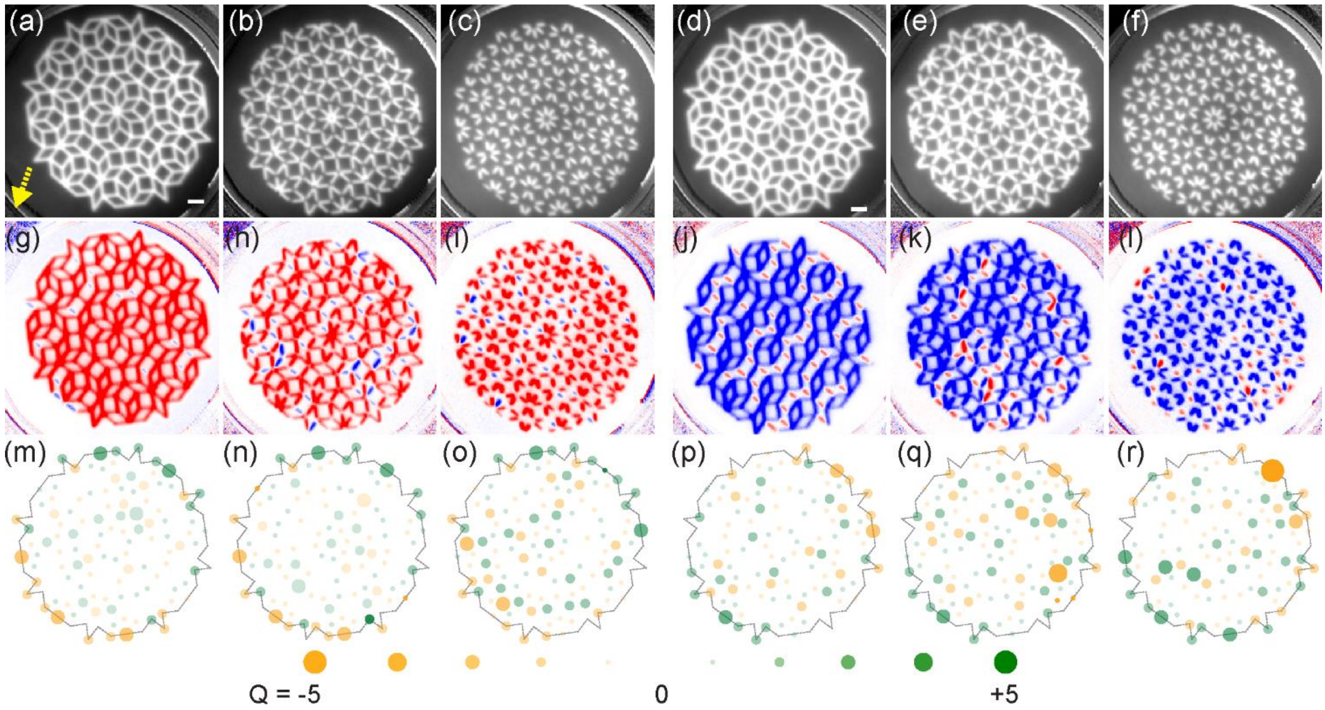

Supplementary Figure 5. PEEM topography image for (a) ATA, (b) ATB, and (c) ATC, respectively in the initial state corresponding to -52 mT → 0 mT and for (d) ATA, (e) ATB, and (f) ATC in the final state corresponding to 52 mT → 0 mT. Bright (dark) regions correspond to magnetic (non-magnetic) regions. The scale bar corresponds to 1 µm. The arrow represents the field direction. The X-ray direction was misaligned by 22° to optimize the detection of nanobars of all the different orientations. (g) - (l) corresponding XMCD data of topography images shown in (a) – (f). Blue (red) colors represent magnetization parallel (opposite) to the X-ray direction. Blue color indicates a reversed nanobar. Notice the weak contrast of nanobars whose orientation deviates from the X-ray direction. (m) – (r) CM analysis of XMCD experimental data shown in (d) – (f). The green and orange filled circles at the vertices represent positive and negative vertex charges, respectively. Here the circles with smallest and largest diameter represent charge $Q = 0$ and $|Q| = 5$, respectively.

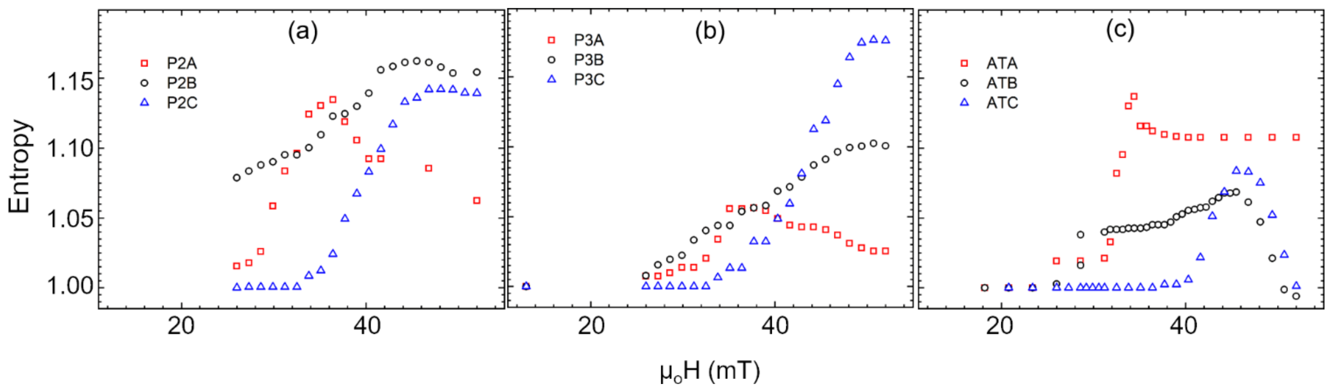

Supplementary Figure 6. (a) Shannon entropy analysis (also known as the degree of randomness) of our analyzed XPEEM images for (a) P2, (b) P3, and (c) Ammann AMQs. The Shannon entropy is defined as -Σ ρ log ρ, where ρ is the normalized (with respect to the initial state) histogram counts of the greyscale image for every XMCD analyzed image. A high value in Shannon entropy represents a high amount of information in an analyzed XPEEM image. Notice that the magnetic field corresponding to a peak in entropy coincides with the field at which a maximum in flux closure loops appears (Figs. 4 and 5 in the main text).

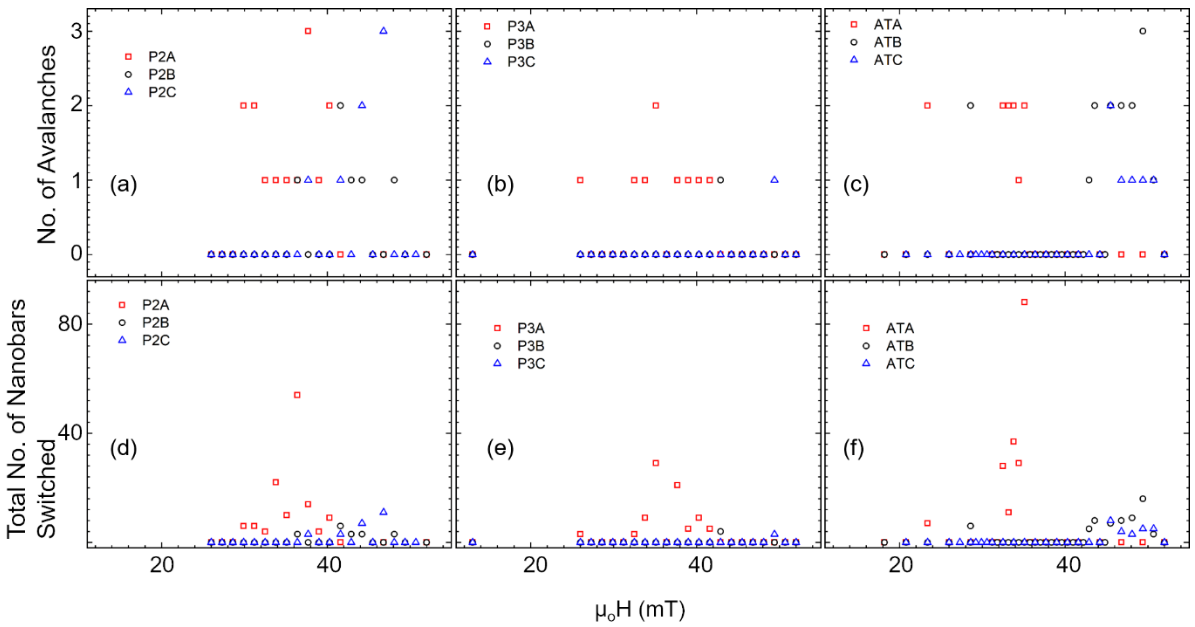

Supplementary Figure 7. Number of avalanches in (a) P2, (b) P3, and (c) Ammann AMQs at every field step. (e) - (f) Total number of nanobars switched for the total number of avalanches shown in (a) - (c), respectively. We define an avalanche when at least three nanobars that are next to one another take part in the reversal process.

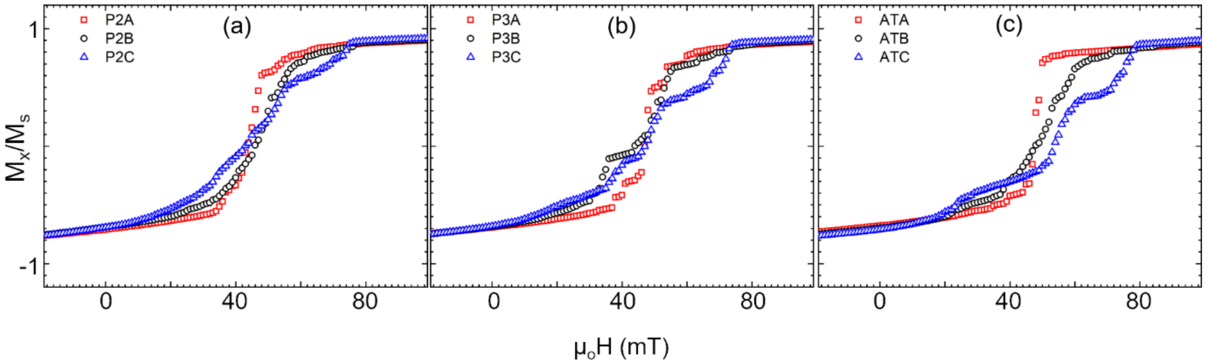

Supplementary Figure 8: Simulated magnetization reversal for (a) Penrose P2, (b) Penrose P3, and (c) Ammann quasicrystal tiling for magnetic field sweep from -1.2 T tesla to +1.2 T. Notice the narrower switching regime for inter-connected P2A, P3A and ATA arrays indicating that vertices in nanobars play important role in magnetization reversal.
